# Three Cases of Presentation of the Face to the Sacrum, Successfully Treated without Turning

**Published:** 1847-01

**Authors:** T. Van Valzah

**Affiliations:** Mifflinburg, Pennsylvania


*Three cases of presentation of the Face to the Sacrum, successfully treated without turning.* By T. Van Valzah, M.D., of Mifflinburg, Pennsylvania.

Mrs. Shively, on the 12th day of February, 1828, sent for me in great haste, on account of a very profuse hemorrhage. When I entered the room, I found her lying on her back near the bedside. With as little delay as possible I made an examination, and found the face low in the hollow of the sacrum. Under these unfavourable circumstances, according to the directions of Dr. Dewees, I determined on turning and delivering by the feet. So without changing her position, I directed an assistant to support her left knee, and supporting the right one myself, I introduced my right hand into the vagina, and placing two fingers on each side of the nose, having the forehead in the hollow of my hand, I raised the whole head up out of the pelvis, and as it became disengaged, by a very little traction with the points of my fingers, the chin fell back towards the sternum, and fortunately at that instant the uterus began to contract with great force. The head being now in the first position I withdrew my hand as it advanced, and the whole cranium was born with the same pain, and the body with the next. The placenta was thrown off in about fifteen or twenty minutes. The mother and child both did well.

April 13th, 1842. Mrs. Beckley, residing in Mifflinburg, sent for me in the evening. On examination I found the face coming down with the chin in the hollow of the sacrum, the os uteri but partially dilated. Encouraged by my success in the treatment of the former case, I now awaited the dilating process very attentively for about two hours, when it became sufficiently great to operate. I then had my patient placed on her back near the bed stock, and directing an assistant to support the left knee, while I supported the right myself, I introduced my hand into the vagina, placing two fingers on each side of the nose, having the forehead in the hollow of my hand, I disengaged the head from the pelvis by raising it up, at the same time directing the chin towards the sternum. The head being now in the first position, I withdrew my hand as it advanced. The labour progressed speedily and safely. The mother and child both did well.

April 21st, 1842. Mrs. Kleckner, about to be confined the eighth or ninth time, sent for me in the afternoon. On making an examination, "per vaginam," I found this also to be a face presentation, and in every respect similar to that of Mrs. Beckley, which occurred but eight days before. It is sufficient to say that I treated this case precisely as I did Mrs. Beckley. Mother and child are both living and well at this time.

